# Cracking the Code: Predicting Tumor Microenvironment Enabled Chemoresistance with Machine Learning in the Human Tumoroid Models

**DOI:** 10.21203/rs.3.rs-5159414/v1

**Published:** 2025-04-21

**Authors:** Geeta Mehta, Michael Bregenzer, Pooja Mehta, Kathleen Burkhard

**Affiliations:** University of Michigan; University of Michigan; University of Michigan; University of Michigan

**Keywords:** ovarian cancers, high grade serous carcinoma, organoid, cell composition, drug response, chemoresistance, spheroids, molecular subtype, HGSC

## Abstract

High-grade serous tubo-ovarian cancer (HGSC) is marked by substantial inter- and intra-tumor heterogeneity. The tumor microenvironments (TME) of HGSC show pronounced variability in cellular make-up across metastatic sites, which is linked to poorer patient outcomes. The influence of cellular composition on therapy sensitivity, including chemotherapy and targeted treatments, has not been thoroughly investigated. In this study, we examined the premise that the variations in cellular composition can forecast drug efficacy. Using a high-throughput 3D in vitro tumoroid model, we assessed the drug responses of twenty-three distinct cellular configurations to an assortment of five therapeutic agents, including carboplatin and paclitaxel. By amalgamating our experimental findings with random forest machine learning algorithms, we assessed the influence of TME cellular composition on treatment reactions. Our findings reveal notable disparities in drug responses correlated with tumoroid composition, underscoring the significance of cellular diversity within the TME as a predictor of therapeutic outcomes. However, our work also emphasizes the complex nature of cell composition’s influence on drug response. This research establishes a foundation for employing human tumoroids with varied cellular composition as a method to delve into the roles of stromal, immune, and other TME cell types in enhancing cancer cell susceptibility to various treatments. Additionally, these tumoroids can serve as a platform to explore pivotal cellular interactions within the TME that contribute to chemoresistance and cancer recurrence.

## Introduction

Most ovarian cancer patients are treated with standard non-personalized treatment regimen of neoadjuvant chemotherapy (NACT) comprising of platinum and taxane therapy followed by surgical debulking^1^. While this treatment regimen is often initially effective, most patients experience relapse with the development of chemoresistance leading to high mortality^2,3^. Personalized medicine is a promising approach to improve patient outcomes wherein clinical management is based on the specific characteristics of each individual tumor and patient. However, high grade serous ovarian cancer (HGSC) has a highly heterogeneous clinical response and a paucity of prognostic factors with which patients can be stratified. Factors like *BRCA1/2* or *CCNE1* mutations can aid in directing clinical management, but ovarian cancer patients are still plagued by frequent development of drug resistant disease^4,5^. As a result, there has been a considerable effort to define molecular subtypes of HGSC in order to improve prognosis predictions, and more effectively determine clinical management course of action to improve outcomes^6–11^.

In this quest, a seminal study identified four molecular subtypes of HGSC (mesenchymal, immunoreactive, proliferative, and differentiated) in microarray analysis of 285 serous and endometriod tumors^10^. The mesenchymal subtype with high stromal and low immune signature corresponded with the worst clinical outcomes, while high immune and differentiated signatures were associated with more favorable outcomes^10^. Since the publication of this report, over the years these molecular subtypes have been validated by some studies while others have developed unique molecular classifications of HGSC into similar, yet different subtypes^6–12^. However, the HGSC molecular subtyping has not been implemented clinically, in part because the specific subtype markers are not statistically significant across all studies^3^. Yet, the promise of using molecular subtyping in HGSC has been demonstrated, for example in a retrospective study of the effectiveness of anti-angiogenic therapy bevacizumab^13^. By molecular subtyping HGSC, bevacizumab conferred a greater overall benefit in the two subtypes with worst prognosis (mesenchymal and proliferative), thereby indicating that subtype specific treatments may improve clinical outcomes^13^. This example highlights the potential of molecular subtype signatures to direct clinical management. A better understanding of the role of the nuanced tumor microenvironment cellular composition (i.e., high stromal, high immune, etc.) in patient drug responses to various drugs could enhance the clinical value of HGSC molecular subtyping efforts. Machine learning models are poised to accomplish this goal and provide additional context for developing new treatments and direct the effective administration of current treatments.

Machine learning models are uniquely suited to elucidate the role of nuanced non-tumoral cellular composition in drug response and chemoresistance due to their ability to identify meaningful patterns in complex multi-dimensional datasets^14–16^. As an example, Yu et al. developed a machine learning model trained on proteomic profiles of 130 ovarian serous carcinoma patients to predict response to platinum therapy using various supervised machine learning algorithms and proposed the key pathways involved in platinum resistance with their bioinformatic approach^17^. In another study, explicit mathematical models with a machine learning framework were combined in order to identify candidate combinations of existing therapies and test mechanistic hypotheses for improving treatment efficacy^18^. Together, these and other examples of deep learning and neural network techniques in predicting drug responses highlight the value of interpretable machine learning models in clinical and translational applications^19–24^.

Random forest is one of the types of machine learning approach with high interpretability for biological applications^14,16,25–27^. This approach not only achieves accurate predictions, but is also robust for non-parametric data, outliers, and over-fitting^28^. Furthermore, random forest enables measurement of feature importance to gain information on the process used to generate the model, or what the model ‘learned’ about the data. Therefore, random forest models are a popular choice in biomedical machine learning due to these advantages and have resulted in many successful models^29^.

In order to address the unmet need for improved molecular subtyping in HGSC and to clarify the role of the tumor microenvironment cellular composition in response to treatment, we utilized a combination of *in vitro* drug screening of heterogeneous tumoroids and easily interpretable machine learning techniques in this report. We hypothesize that tumoroids engineered with different cellular compositions (all including tumor cells) will respond differentially to drug treatments. Furthermore, we expect the cell composition of each tumoroid to be used to make predictions of drug response and to gain insights into key cell-cell relationships in evolving chemoresistance. Finally, we expect that the findings from the integrated tumoroid and machine learning model presented in this report will translate to effective molecular subtyping of patient-specific drug responses, in order to improve HGSC response and cure rates.

## Results

### Tumoroids derived from different tumor microenvironment cell compositions respond differentially to 5 different chemo- and targeted therapies.

**Figure 1.**
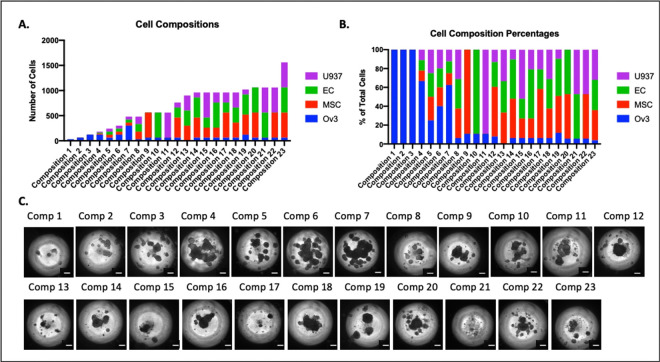
Heterogeneous high grade serous ovarian tumoroids were created with 23 different cell compositions. **A**) Bar graph showing the total cell number in each tumoroid composition distributed between high grade serous cell line OVCAR3 (blue), mesenchymal stem cells (MSCs) (red), endothelial cells (ECs) (green), and U937 monocytes (purple). **B**) Bar graph showing the percentage of each cell type in each composition with OVCAR3 represented in blue, MSCs in red, ECs in green, and U937 monocytes in purple. **C**) Phase contrast image of tumoroids taken on day 5, made with each cell composition showing heterogeneous size and morphology. Scale bar = 200mm.

HGSC are comprised of multiple cell types, including epithelial cancer cells, mesenchymal cancer cells, carcinoma-associated mesenchymal stem cells, microvascular endothelial cells and macrophages, each of which support tumor cells in different contexts^30–32^. The mesenchymal subtype signature is strongly expressed by the fibroblasts and mesenchymal stem cells, while the immunoreactive signature features myeloid, T cells, and NK cells. Meanwhile, epithelial cells highly express differentiated signature and to a lesser extent express proliferative signature^33^. In order to investigate the impact of non-tumoral cellular composition on the HGSC chemosensitivity, 23 tumoroids of different cell compositions (Fig. 1A–C, Table 1) were fabricated. Differential morphologies of tumoroids were observed for each of the 23 compositions (Fig. 1C). The responses of cancer cells in tumoroids to conventional chemotherapies (carboplatin and paclitaxel) as well as targeted biologic treatments (PACMA31, N773, and SC144) were tested (Fig. 1A–C, Table 1) and assessed for viability using the MTS assay following 48 hours of drug treatment. Three biologic targeted treatments utilized in this study were: protein disulfide isomerase (PDI) inhibitor (PDIi), PACMA31^34^; IL-6/STAT3 inhibitor, SC144^35–37^; and E2F/RUNX2 inhibitor (compound N773).

**Figure 2.**
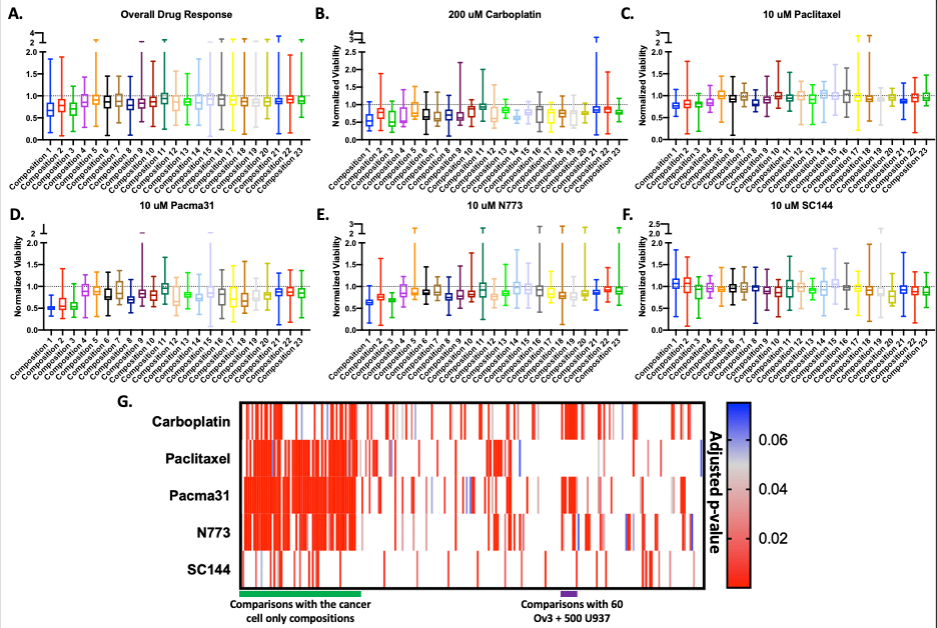
Heterogenous high grade serous ovarian tumoroids with different cell composition have statistically significant differences in drug treatment response. **A)** Box and whisker plot of averaged normalized viability for each tumoroid cell composition across all treatment conditions showing overall resistance to treatment. Box and whisker plots showing resistance of each tumoroid composition to **B**) carboplatin, **C**) paclitaxel, **D**) PACMA31, **E**) N773, and **F**) SC144. **G**) Heatmap of adjusted p-values for comparisons between all conditions determined via one-way ANOVA and Tukey’s post hoc analysis. Red indicates most significant (p<0.05), grey indicates borderline significance (p=0.05), and blue and white indicate not significant (p>0.05).

Within each drug treatment, there were significant differences in cancer cell viability between tumoroid cell compositions (Fig. 2A–G). In Fig. 2G, the most significant differences were clustered in the left half of the heatmap, corresponding to comparisons of compositions 1, 2, and 3 (the cancer cell only conditions) with the other 20 compositions that also contained non-tumor cells. Paclitaxel, PACMA31, and N773 treatment yielded the most significant differences in viability. Interestingly, a similar cluster of significant differences exists in both the carboplatin and PACMA31 treatment group (Fig. 2G). Upon examination, these clusters correspond to the increased chemoresistance of composition 11 (with macrophages and cancer cells) compared to compositions 12–20 comprising of three or four cell types (all contain MSCs and endothelial cells) (see Fig. 1A, B, Table 1).

We characterized the chemoresistant tumoroids as those with highest average pooled viability after drug treatments. Notably, the top five chemoresistant tumoroid compositions were compositions 11, 15, 5, 22, and 23 in order of most viable to least (Table 2). Interestingly, 4 out of the 5 most resistant compositions on average included 500 U937 (myeloid cells). Similarly, we characterized the chemosensitive tumoroids as those with the lowest average pooled viability after drug treatments. The most chemosensitive tumoroids after all drug treatments were compositions 19, 8, 2, 1, and 3 in order of most viable to least (Table 3). We noted that the three most sensitive tumoroid compositions on average were the only three conditions generated with cancer cells only.

However, the compositions with the greatest resistance and sensitivity to treatment varied depending on the treatment (Tables 2 and 3). Briefly, compositions 11, 21, 22, 5, and 13 were the most resistant to carboplatin treatment while compositions 19, 7, 14, 3, and 1 were the most sensitive to carboplatin. Notably, composition 13 contained no cancer cells. Contrarily, the most resistant compositions to paclitaxel were compositions 14, 5, 10, 16, and 15, two of which contained 500 ECs (Table 2). Compositions 4, 8, 2, 1, and 3 were the most sensitive to paclitaxel, again including the three tumoroids comprised of cancer cells only. PACMA31 was least effective against and saw the most resistance from compositions 11, 7, 4, 5, and 15. Compositions 18, 8, 2, 3, and 1 were the most sensitive to PACMA31 treatment. Compositions 14, 11, 16, 15, and 22 were most resistant to N773 treatment, including three conditions with 500 U937(myeloid cells). Compositions 19, 8, 2, 3, and 1 were the most sensitive to N773 treatment. Finally, compositions 15, 1, 2, 12, and 7 were most resistant to SC144 treatment. Compositions 19, 23, 3, 10, and 20 were most sensitive to SC144. Interestingly, among the least impacted compositions for SC144 were cancer cell only compositions with 30 and 60 OVCAR3 cells respectively (Table 3). Therefore, we observed differential responses to both chemo- and targeted therapies in the 23 tumoroid compositions, with 3 cancer cell only compositions being the most sensitive to drug treatments. These data highlight the potential clinical application of tumoroids in predicting responses to targeted therapies.

### “Mesenchymal” tumoroids respond differentially to carboplatin and paclitaxel

**Figure 3.**
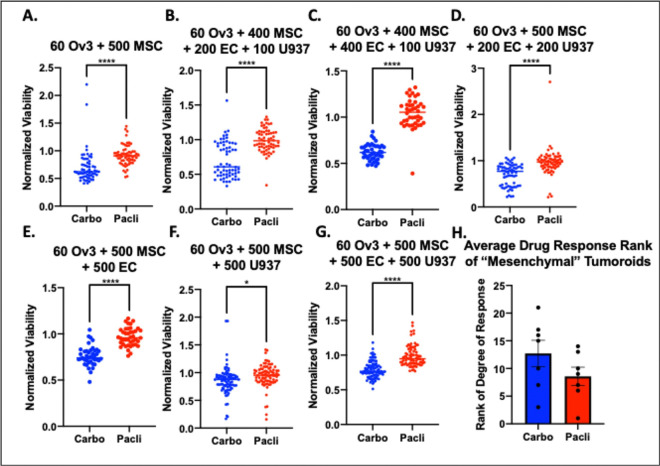
Comparison of carboplatin and paclitaxel response among high grade serous ovarian tumoroids with the highest number of mesenchymal stem cells, termed “mesenchymal” tumoroids. **A-G**) Average normalized viability of each mesenchymal tumoroid following treatment with carboplatin or paclitaxel. **H**) Average resistance rank of each mesenchymal tumoroid to carboplatin (blue) or paclitaxel (red) compared to all other tumoroid compositions.

Reports in the literature support that mesenchymal subtypes are more sensitive to taxane (paclitaxel) therapy and more resistant to platinum based therapies^38^. Mesenchymal subtype signatures have also been linked to the presence of mesenchymal cells, as opposed to more mesenchymal cancer cell populations^32^. Therefore, in order to draw a first pass clinical parallel with our tumoroid models, we compared the drug response of 7 “mesenchymal” tumoroid compositions (compositions 9, 12, 14, 17, 20, 22, and 23) characterized by ≥400 MSCs, to carboplatin treatment versus paclitaxel treatment (Fig. 3A–G). This comparison yielded the opposite relationship than expected from literature, with carboplatin being more effective in the selected “mesenchymal” tumoroids compared to paclitaxel treatment.

To combat the relative effectiveness of carboplatin versus paclitaxel dosing, we next examined the relative ranking of the “mesenchymal” tumoroids compared to all other compositions treated with carboplatin or paclitaxel (Fig. 3H). We expected that mesenchymal tumoroids would rank in the most resistant compositions among the carboplatin treatment group and in the least resistant compositions among the paclitaxel treatment group. Surprisingly, the tumoroids with the most MSC were more sensitive to carboplatin, with an average ranking of 12.7 (in the bottom half of all compositions), while the mesenchymal tumoroids were more resistant to paclitaxel, with an average rank of 8.6 (in the top half of all compositions). Additionally, the most resistant composition to paclitaxel, composition 14, is a mesenchymal tumoroid. Of note, composition 22 was the only mesenchymal tumoroid that showed the expected trend (third most resistant composition to carboplatin and the thirteenth most resistant to paclitaxel). We also noted that the composition without cancer cells, composition 13, also followed the expected mesenchymal subtype trend being the fifth most resistant to carboplatin and the fifteenth most resistant to paclitaxel. Therefore, the mesenchymal tumoroids with higher numbers of mesenchymal stem cells, were sensitive to carboplatin and resistant to paclitaxel, which is opposite of literature report for mesenchymal subtype signature.

### Machine Learning Models Predicted Drug Responses Based on the Tumoroid Cellular Composition.

To investigate the utility of the tumor microenvironment cell composition in predicting treatment response, random forest models were created to predict tumoroid response to each drug based on cell composition. The primary purpose of the machine learning approach was to use it as a tool to establish potential relationships between cell composition features and response to drug treatment, not necessarily to generate highly accurate prediction models. The median normalized absorbance value from the MTS viability assays for each drug treatment was used to determine ‘high’ or ‘low’ response to the drug.

**Figure 4.**
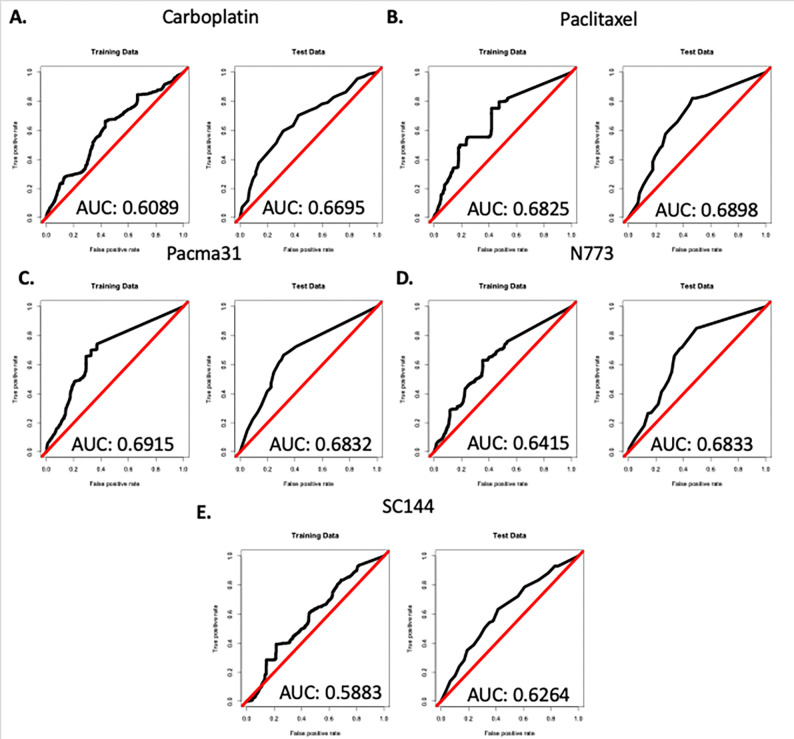
Random forest model performed better than random predictors at predicting drug responses to each treatment for all tumoroid cell compositions. Receiver operating characteristic curves (ROCs) for the training (left) and test (right) set for random forest model generated to predict response to **A**) carboplatin, **B**) paclitaxel, **C**) PACMA31, **D**) N773, and **E**) SC144.

The random forests had variable effectiveness at predicting response to different drugs. The worst model (predicting SC144 response) had an AUC of 0.5883 for the training set and 0.6264 for the test set (Fig. 4E). Contrarily, the most effective random forest model had an AUC of 0.6915 for the training set and an AUC of 0.6832 on the test set when trained to predict PACMA31 response (Fig. 4C). Paclitaxel (training AUC: 0.6825; test AUC: 0.6898; Fig. 3B) and N773 (training AUC: 0.6415; test AUC: 0.6833; Fig. 4D) predictions were the second and third most accurate. The carboplatin model was the second to last most effective with a training AUC of 0.6089 and a test AUC of 0.6695 (Fig. 4A). Each model performed better than a random predictor and was relatively consistent between the training and test sets indicating lack of overfitting. Training set AUCs ranged from 0.5883 to 0.6915, indicating moderate performance. Interestingly, the three most accurate prediction models (PACMA31, paclitaxel, and N773) were based on treatments with the greatest significant difference in viability between experimental conditions with and without non-tumoral cells (Fig. 2G).

### Random Forest Models Predicted Most Important Parameters for Individual Drug Response.

**Figure 5.**
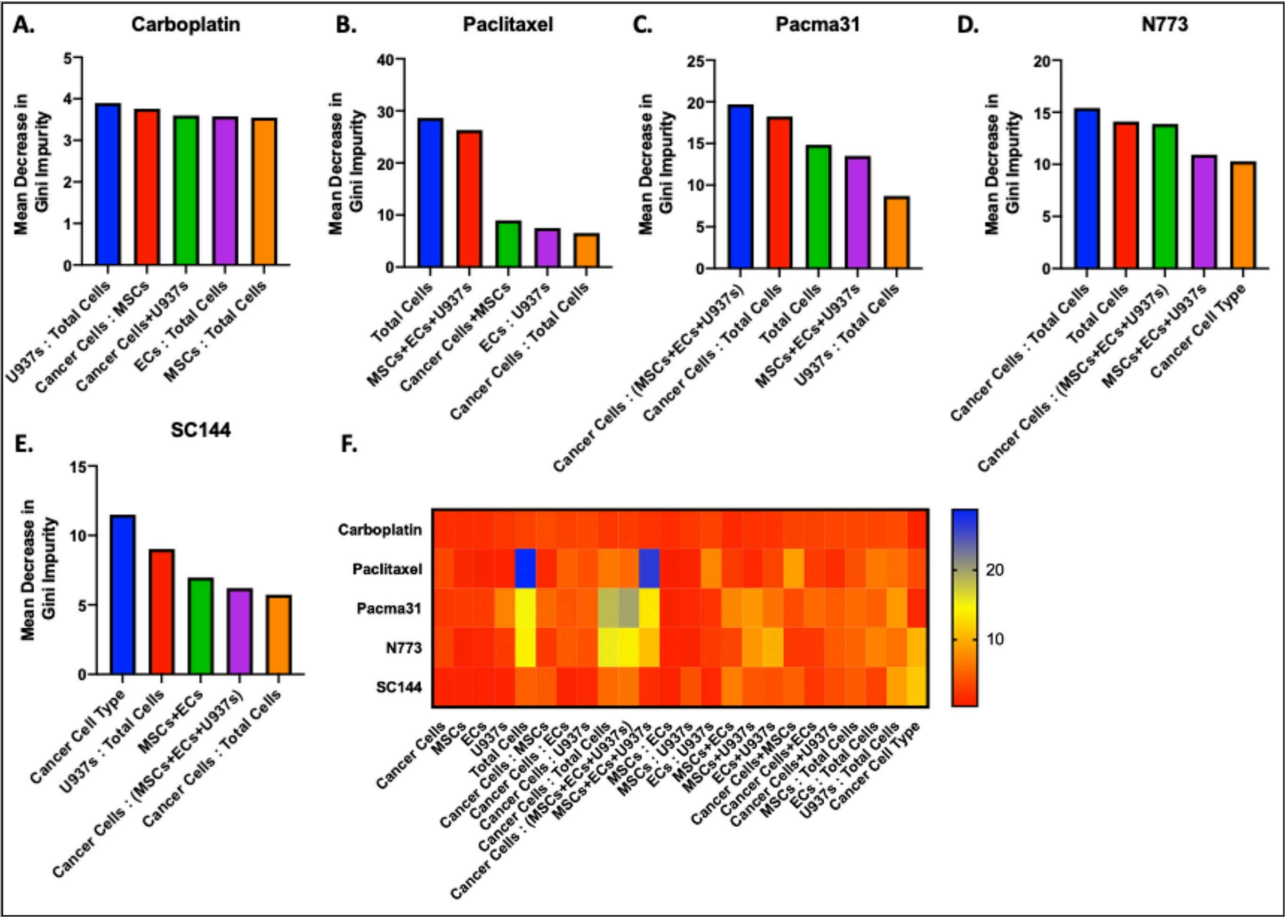
Random forest model provided easily accessible variable importance measures to facilitate interpretation of results. Top 5 most influential parameters in predicting drug response to **A**) carboplatin, **B**) paclitaxel, **C**) PACMA31, **D**) N773, and **E**) SC144. **F**) Heatmap of the importance measures of all parameters for each drug prediction model across all tumoroid cell compositions.

We evaluated the importance of each tumoroid composition parameter in making predictions for each drug response. None of the random forest models prioritized the number of a single cell type in making predictions. The carboplatin model placed relatively equivalent importance on most of its parameters, but still put the most weight on the proportion of the tumoroid made up of U937 (myeloid cells). The proportion of the tumoroid made up of endothelial and mesenchymal stem cells respectively were also in the top 5 most important parameters in the carboplatin model (Fig. 5A). The paclitaxel response predictor placed the most importance by far on the total number of cells and the total number of all tumor and non-tumor cells plated at the start of experiment (Fig. 5B). As the total number of tumor microenvironment cells is highly influential in the total number of cells (more non-tumoral cells inherently means more total cells), these two parameters are heavily overlapping. PACMA31 predictions placed the most importance on the ratio of cancer cells to TME cells and to total cells respectively (Fig. 5C). N773 model placed a similar weight on the ratio of cancer cells to total cells and the total number of cells alone. Finally, SC144 model placed the greatest importance on the cancer cell type (OVCAR3 sorted for cancer stem-like cells (CSC) markers^36,39–41^ ALDH and CD133, compared to unsorted OVCAR3), as an experiment was conducted with sorted ovarian CSC as opposed to unsorted OVCAR3. The SC144 model also placed high importance on the proportion of myeloid cells in the tumoroids (Fig. 5E). Figure 5F shows a heatmap of the importance value of each parameter for each drug response prediction model, revealing a clear pattern of the importance with the proportion of cancer cells and TME cells in each tumoroid being critical parameters. The heatmap also reveals moderate importance placed on various cell – cell relationships whereas the parameters that considered only individual cell type numbers were among the least important for all models.

**Figure 6.**
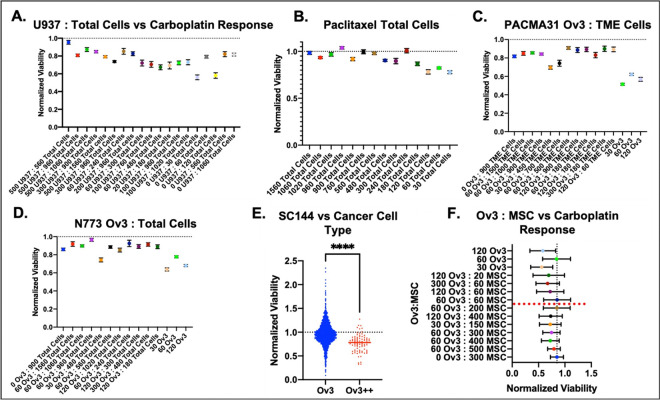
Normalized viability versus the important features for each random forest model reveal cell composition dynamics in drug response prediction. The most important features in predicting response to **A**) carboplatin, **B**) paclitaxel, **C**) PACMA31, **D**) N773, and **E**) SC144 versus normalized viability. **F**) Plotting the second most important feature in predicting carboplatin response versus normalized viability reveals the expected relationship between presence of mesenchymal cells and resistance to carboplatin (higher ratios of MSCs to OVCAR3 is associated with greater resistance to carboplatin).

Breaking down the most important parameters for each model and plotting the important parameter versus the treatment response showed the relationship between each tumoroid composition variable and the treatment response (Fig. 6A–E). In general, there appears to be an inverse relationship between drug response and total number of non-tumoral cells, which is highly correlated with the total cell number. For example, PACMA31 appears to be most effective when the ratio of cancer cells to mesenchymal stem cells, endothelial and myeloid cells is high (i.e. there are no added TME cells) (Fig. 6C). Interestingly, the SC144 prediction model placed the most importance on the type of OVCAR3 cells used, as tumoroids made with CSC sorted OVCAR3 were more susceptible to SC144. Moreover, when examining the second most important parameter in predicting response to carboplatin, low ratios of OVCAR3 to MSC (a higher proportion of MSC than cancer cells) resulted in generally greater resistance to carboplatin treatment (Fig. 6F). Therefore, the random forest models revealed the most important parameters that were instrumental in predicting the individual drug responses.

## Discussion

The cellular composition of the TME has previously been reported in driving differential treatment responses between HGSC molecular subtypes^30,32,33^. However, there is an incomplete understanding of the role of more nuanced differences in non-tumoral cellular composition on treatment outcomes. In this work, we leveraged a 3D, tunable heterogeneous tumoroid platform to screen the drug responses of tumoroids representing 23 distinct compositions of tumor cells, mesenchymal stem cells, endothelial and myeloid cells. Screening data was subsequently used to generate random forest models to predict drug response based on tumoroid cell composition.

The most significant differences in treatment response were found between tumoroid compositions with only cancer cells and those with added tumor microenvironment non-tumoral cells. The three most sensitive compositions contained only cancer cells, supporting the role of TME cells in conferring chemoresistance, as expected based on the literature^40,42–44^. Differences in drug response between compositions with varying numbers of TME cells suggest composition-dependent chemoresistance effects. From a clinical application perspective, this could indicate that those three drugs would be relatively less effective in tumors with high stromal content.

Treatments with paclitaxel, PACMA31, and N773 yielded the most significant differences in viability, potentially indicating that their respective mechanisms of action are more attenuated by non-tumor cells, than the mechanisms leveraged by carboplatin and SC144. Significant differences were identified in cluster of tumoroid compositions treated with carboplatin and PACMA31, with increased chemoresistance of composition 11 compared to compositions 12–20. This cluster of differences included the significant difference between composition 11 and composition 15, which both contain 500 myeloid cells, refuting the rationale that myeloid cells are solely responsible for chemoresistance observed in composition 11. It is possible that carboplatin and PACMA31 are negatively influencing viability in composition 15 through effects on the MSCs and/or ECs. Alternatively, it is possible that the MSCs and/or ECs are interacting with the cancer cells and/or myeloid cells to attenuate the chemoresistant effect observed in composition 11. The molecular mechanism driving this response requires further experimentation but poses some interesting questions with potential translational implications. We additionally observed that paclitaxel was least effective against compositions 14, 5, 10, 16, and 15, two of which contained 500 ECs, potentially indicating a connection between ECs and resistance to paclitaxel (Table 3). This is contrary to previous reports of anti-angiogenic and endothelial-specific effects of paclitaxel^45–47^. However, it is possible that ECs are conferring chemoresistance through indirect mechanisms such as the modulation of tumoroid extracellular matrix^48^.

After generating random forest models for each drug treatment, we found that the model predicting SC144 response had the worst performance but was still better than a random prediction model. The tumoroid compositions that were least impacted by SC144 contained cancer cell only compositions. SC144 inhibits gp130 through binding resulting in gp130 phosphorylation and deglycosylation and ultimately abrogates STAT3 phosphorylation and subsequent downstream gene activation^49^. Through these mechanisms, SC144 was previously shown to be effective against ovarian cancer cell lines *in vitro* and *in vivo*^49^. However, in our tumoroid platform, overall response across all conditions was low as indicated by sparse significant differences between experimental compositions. This low overall response perhaps suggests the need for increased doses in our system to identify true responses. More robust drug responses obtained with higher concentration treatment or longer duration treatment could also reduce overlap in response values between compositions, potentially leading to more accurate model predictions.

When we examined the response of “mesenchymal tumoroids” (those with at least 400 MSCs) to carboplatin and paclitaxel effectiveness, we found that carboplatin was more effective in the selected “mesenchymal” compositions compared to paclitaxel treatment. This was contrary to expectation. Although, like SC144, paclitaxel had low overall response, suggesting the potential need for higher doses or longer treatments to ensure that treatment effects are not being obscured. This finding could also be due to a number of factors including the crude nature with which we defined “mesenchymal” tumoroids. It is possible that some other tumoroid compositions would be classified as a mesenchymal subtype if they were to be sequenced, even with lower numbers of MSC at the start of the experiment. In fact, previous work in our lab compared the gene expression in patient-specific tumoroids made with composition 18 (60 patient specific tumor cells + 300 MSC + 300 EC + 300 PBMC) versus matched patient-specific spheroids made with only 60 patient tumor cells. In this work, patient specific tumoroids were found to be more resistant to carboplatin treatment and to have a transcriptome reflective of the mesenchymal subtype. From this, we may infer that composition 18 in this analysis, and perhaps other compositions, could have been justifiably considered as “mesenchymal” tumoroids. However, in the absence of sequencing data to group each composition into appropriate molecular subtypes, this possible explanation remains conjecture. Furthermore, while mesenchymal subtypes have been attributed to the presence of mesenchymal cells, the subtype signature may also be influenced by other cell types in the stroma making our crude definition of a “mesenchymal” tumoroid less meaningful.

We found that paclitaxel was least effective against compositions 14, 5, 10, 16, and 15, all of which contained 500 ECs. This is not in line with previous reports of anti-angiogenic effects of paclitaxel but might be related to ECs conferring chemoresistance through modulation of tumoroid extracellular matrix. The response to SC144 was low across all conditions, suggesting a need for higher doses. Carboplatin was more effective in “mesenchymal” tumoroids (those with at least 400 Mesenchymal Stem Cells) compared to paclitaxel, which had low overall response. Only composition 22 was the most representative of the “mesenchymal” molecular subtype. The three most accurate prediction models were those that had the most significant differences in response between cancer cell only conditions and compositions with added non-tumoral cells, indicating that these three drugs would be less effective in tumors with high stromal content. Overall, training set AUCs ranged from 0.5883 to 0.6915, indicating moderate performance. However these values are in line with those of previous drug prediction models generated based on protein (AUC: ~0.58–0.64 for various machine learning algorithms)^17^ or molecular features (AUC: ~0.56–0.76 for a Deep Neural Network model)^50^ demonstrating the potential of predictions made based solely on cell composition.

The results of this study highlight the importance of considering complex relationships between cell types in predicting drug response. The random forest models did not use the quantity of a single cell type as a significant factor, but rather more complex parameters that considered multiple cell types. One example was the relationship between PACMA31 response and the ratio of cancer cells to MSCs, ECs, and U937s, where a high ratio of cancer cells was found to be more effective. Another example was the importance of the type of OVCAR3 cells used in the experiment for predicting SC144 response, where tumoroids made with sorted OVCAR3 CSCs were more susceptible to SC144^51–53^, potentially due to SC144’s ability to prevent STAT3 activation which is known to be involved in CSC maintenance. These findings emphasize the need for complex in vitro models with more than two cell types for drug screening applications and the limitations of human observation alone in understanding the nuanced aspects of TME-conferred chemoresistance.

Lastly, examining the relationships between important parameters and drug response showed the direction between each parameter and treatment response. A more nuanced analysis, using the ratio of cancer cells to MSCs, showed the expected carboplatin response trend of increased resistance in more mesenchymal tumors. This highlights the importance of cell-cell relationships in drug response stratification. Machine learning models have the potential to predict therapy response based on cell composition and evaluate the importance of each parameter. Although the models have moderate accuracy, they can be improved with more physiologically relevant tumoroids (with matched cancer-associated stromal and immune cells) and higher concentration or longer duration drug treatments to obtain more robust drug responses.

## Conclusions

Recent work has suggested that the stromal cells can impact molecular subtyping classifications and thus may have prognostic significance^31,32,54^, however the exact role of nuanced differences in stromal composition in drug response is still unclear. Furthermore, current drug treatment prediction models rely on bulk-omics measurements or expensive imaging techniques, which are often limited in sample size and only exist for previously administered therapies^16,17,27,55–58^, limiting their utility in predicting response to novel therapies. In this work, we developed an in vitro model system to generate tumoroids with different cell compositions, and tested drug response using a high throughput hanging drop plate platform. Random forest models were used to predict drug response based on tumoroid composition. The results showed significant differences in drug response based on tumoroid composition, supporting the importance of the cellular composition of the tumor microenvironment (TME) in predicting therapy response^40,59–63^. Yet, it highlights the importance of nuanced differences in cell composition that may obscure the drug responses. This work provides proof of concept for using tumoroids with different compositions to investigate the role of TME cells in resistance to therapies.

## Materials and Methods

### Cell Culture and Materials.

Epithelial ovarian cancer cells (OVCAR3: American Type Culture Collection, ATCC), were cultured in RPMI 1640 (Gibco) supplemented with 10% fetal bovine serum (FBS: Atlanta Biologics) and 1% antibiotics and antimycotics. Human adipose-derived mesenchymal stem cells (haMSCs: Lonza PT-5006) were cultured in Adipose-derived Stem Cell Basal Medium (Lonza) supplemented with 10% FBS and 1% antibiotics and antimycotics as well as 2 mM L-glutamine (Gibco). Human umbilical vein endothelial cells (HUVECs) were a donation from Lonza and were cultured in Endothelial Basal Medium-2 (EBM-2 [AKA EGM-2]: Lonza). U937 monocytes were purchased from ATCC and were cultured in 1640 RPMI supplemented with 10% heat inactivated FBS and 1% antibiotics and antimycotics. Tumoroid cultures were formed in 384-well hanging drop plates in Tumoroid Medium (TM) (2X SFM: EBM-2 (5:1) + 20 μM ROCK inhibitor). See **Supplemental Table 1** for detailed composition.

Ovarian CSCs were isolated from the high grade serous ovarian cancer cell line OVCAR3 as described previously^64^. Briefly, cells were harvested and incubated with ALDEFLUOR reagent (Stem Cell Technologies, Vancouver BC), and CD133 antibody (Milentyi Biotech, San Diego CA), and sorted using flow cytometry for cells positive for elevated ALDH and CD133 positivity. Appropriate DEAB and isotype controls were used for both assays, to determine gate settings as described previously. CSCs were freshly sorted and used to make tumoroids < 24 hours after flow sorting.

To plate tumoroids in 384-well hanging drop plates, each cell type is collected from 2D plates and resuspended in their respective 2D culture medium for counting on a hemacytometer. The cell density of each cell type is calculated and then used to calculate the total volume of that cell suspension that will be needed to obtain the required number of that cell type per each experimental composition. The calculated volume of each cell suspension is then added into a single tube per tumoroid composition, spun down at 800g and resuspended in the appropriate volume of tumoroid medium so that every well plated on the hanging drop plate will receive 20 μL the desired number of each cell type.

Tumoroids were imaged on day 3, 5, and 7 using an epifluorescent Olympus microscope to obtain phase contrast images (4X). On day 3 tumoroids are supplemented with 2 μL of tumoroid medium per well. On day 5, each tumoroid composition was fed with 2 μL of tumoroid medium with no drug as a control or 200 μM carboplatin, 10 μM paclitaxel, 10 μM PACMA31, 10 μM N773, and 10 μM SC144. Each treatment condition contained 20–40 wells as technical replicates. Plates were incubated for 48 hours and imaged on day 7.

To quantify viability, an MTS assay was used. 2 μL of MTS reagent was added to each well and plates were incubated at 37 degrees C. Absorbance was then measured in each well at 2 hour and 4-hour incubation time points. Normalized viability was quantified by averaging all control wells and dividing the absorbance in each well by the control average to obtain viability measurements for each drug in reference to the viability of the control. Drug assays for each tumoroid composition were repeated in 2–12 separate experiments (most compositions were 2–4 replicates, but composition 2 and 18 had 12 and 7 replicates each because they served as frequent control compositions).

### Data Processing and Model Generation.

Normalized MTS viability data was compiled into an excel spreadsheet and coupled with the corresponding input cell composition for a given experiment. To avoid ‘zero values’ that would lead to “N/As” that need to be excluded or imputed in random forest models, a value of 0.001 was added to all cell numbers. Features were generated by calculating various relationships between the four cell numbers obtained at the inception of the experiment.

Compiled data was then grouped by drug treatment and saved in different files that were then read into Rstudio v1.4.1093 with R version 4.1 for use in model generation. The median absorbance value for each drug treatment was calculated and used as a threshold to assign each replicate a ‘high’ or ‘low’ response label. After appending the response label to the dataset, it was converted into the ‘Factor’ datatype for use as the prediction variable. The normalized absorbance values (and any other parameters that were not needed) were then trimmed from the dataset.

The trimmed dataset was then split into a training set (75% of the samples) and a test set (25% of the samples). At this point, the seed was set at ‘123’, in order to facilitate repeatable runs of the model. First the training set was used to optimize the number of trees for the random forest to generate and the number of parameters to consider at each node split. Those optimal values were then used as inputs in the ‘randomForest()’ function with the training set as the dataset to build the model and the test set in the test set slot. After running the model, the variable importance (quantified as mean decrease in gini impurity) was saved, ROC curves were generated, and AUC values were calculated to quantify model performance on the training and test sets for each drug.

## Figures and Tables

**Table 1:** The tumoroid cell compositions are listed with the number of cancer cells, mesenchymal stem cells, endothelial cells, and monocytes in each of the 23 experimental conditions.

	Cancer Cells (OVCAR3)	Mesenchymal Stem Cells (MSC)	Endothelial Cells (HUVEC)	Monocytes (U937)
Composition 1	30	0	0	0
Composition 2	60	0	0	0
Composition 3	120	0	0	0
Composition 4	120	20	20	20
Composition 5	60	60	60	60
Composition 6	120	60	60	60
Composition 7	300	60	60	60
Composition 8	30	150	150	150
Composition 9	60	500	0	0
Composition 10	60	0	500	0
Composition 11	60	0	0	500
Composition 12	60	400	200	100
Composition 13	0	300	300	300
Composition 14	60	400	400	100
Composition 15	60	200	200	500
Composition 16	60	200	500	200
Composition 17	60	500	200	200
Composition 18	60	300	300	300
Composition 19	120	400	400	100
Composition 20	60	500	500	0
Composition 21	60	0	500	500
Composition 22	60	500	0	500
Composition 23	60	500	500	500

**Table 2.** The top 5 most resistant tumoroid compositions for each treatment are listed with their corresponding normalized viability values.

	Most Resistant Tumoroid Cellular Composition for Each Treatment					
Order of Resistance	Overall Drug Response	Carboplatin Drug Response	Paclitaxel Drug Response	PACMA31 Drug Response	N773 Drug Response	SC144 Drug Response
**5** **Most Resistant**	Composition 11 *60 OVCAR3 + 500 U937*	Composition 11 *60 OVCAR3 + 500 U937*	Composition 14 *60 OVCAR3 + 400 MSC + 400 EC + 100 U937*	Composition 11 *60 OVCAR3 + 500 U937*	Composition 14 *60 OVCAR3 + 400 MSC + 400 EC + 100 U937*	Composition 15 *60 OVCAR3 + 200 MSC +200 EC + 500 U937*
**4**	Composition 15 *60 OVCAR3 + 200 MSC +200 EC + 500 U937*	Composition 21 *60 OVCAR3 + 500 EC + 500 U937*	Composition 5 *60 OVCAR3 + 60 MSC + 60 EC + 60 U937*	Composition 7 *300 OVCAR3 + 60 MSC + 60 EC + 60 U937*	Composition 11 *60 OVCAR3 + 500 U937*	Composition 1 *30 OVCAR3*
**3**	Composition 5 *60 OVCAR3 + 60 MSC + 60 EC + 60 U937*	Composition 22 *60 OVCAR3 + 500 MSC + 500 U937*	Composition 10 *60 OVCAR3 + 500 EC*	Composition 4 *120 OVCAR3 + 20 MSC + 20 EC + 20 U937*	Composition 16 *60 OVCAR3 + 200 MSC + 500 EC + 200 U937*	Composition 2 *60 OVCAR3*
**2**	Composition 22 *60 OVCAR3 + 500 MSC + 500 U937*	Composition 5 *60 OVCAR3 + 60 MSC + 60 EC + 60 U937*	Composition 16 *60 OVCAR3 + 200 MSC + 500 EC + 200 U937*	Composition 5 *60 OVCAR3 + 60 MSC + 60 EC + 60 U937*	Composition 15 *60 OVCAR3 + 200 MSC +200 EC + 500 U937*	Composition 12 *60 OVCAR3 + 400 MSC + 200 EC + 100 U937*
**1** **Least Resistant**	Composition 23 *60 OVCAR3 + 500 MSC + 500 EC + 500 U937*	Composition 13 *300 MSC + 300 EC + 300 U937*	Composition 15 *60 OVCAR3 + 200 MSC + 200 EC + 500 U937*	Composition 15 *60 OVCAR3 + 200 MSC + 200 EC + 500 U937*	Composition 22 *60 OVCAR3 + 500 MSC + 500 U937*	Composition 7 *300 OVCAR3 + 60 MSC + 60 EC + 60 U937*

**Table 3.** The top 5 most sensitive tumoroid compositions for each treatment are listed with their corresponding normalized viability values.

	Most Sensitive Tumoroid Cellular Composition for Each Treatment					
Order of Sensitivity	Overall Drug Response	Carboplatin Drug Response	Paclitaxel Drug Response	PACMA31 Drug Response	N773 Drug Response	SC144 Drug Response
**5** **Least Sensitive**	Composition 19 *120 OVCAR3 + 400 MSC + 400 EC + 100 U937*	Composition 19 *120 OVCAR3 + 400 MSC + 400 EC + 100 U937*	Composition 4 *120 OVCAR3 + 20 MSC + 20 EC + 20 U937*	Composition 18 *60 OVCAR3 + 300 MSC + 300 EC + 300 U937*	Composition 19 *120 OVCAR3 + 400 MSC + 400 EC + 100 U937*	Composition 19 *120 OVCAR3 + 400 MSC + 400 EC + 100 U937*
**4**	Composition 8 *30 OVCAR3 + 150 MSC + 150 EC + 150 U937*	Composition 7 *300 OVCAR3 + 60 MSC + 60 EC + 60 U937*	Composition 8 *30 OVCAR3 + 150 MSC + 150 EC + 150 U937*	Composition 8 *30 OVCAR3 + 150 MSC + 150 EC + 150 U937*	Composition 8 *30 OVCAR3 + 150 MSC + 150 EC + 150 U937*	Composition 23 *60 OVCAR3 + 500 MSC + 500 EC + 500 U937*
**3**	Composition 2 *60 OVCAR3*	Composition 14 *60 OVCAR3 + 400 MSC + 400 EC + 100 U937*	Composition 2 *60 OVCAR3*	Composition 2 *60 OVCAR3*	Composition 2 *60 OVCAR3*	Composition 3 *120 OVCAR3*
**2**	Composition 1 *30 OVCAR3*	Composition 3 *120 OVCAR3*	Composition 1 *30 OVCAR3*	Composition 3 *120 OVCAR3*	Composition 3 *120 OVCAR3*	Composition 10 *60 OVCAR3 + 500 EC*
**1** **Most Sensitive**	Composition 3 *120 OVCAR3*	Composition 1 *30 OVCAR3*	Composition 3 *120 OVCAR3*	Composition 1 *30 OVCAR3*	Composition 1 *30 OVCAR3*	Composition 20 *60 OVCAR3 + 500 MSC + 500 EC*

## Data Availability

The data that support the findings of this study are available from the corresponding author, upon reasonable request.
